# Platelet Transfusion – The New Immunology of an Old Therapy

**DOI:** 10.3389/fimmu.2015.00028

**Published:** 2015-02-02

**Authors:** Moritz Stolla, Majed A. Refaai, Joanna M. Heal, Sherry L. Spinelli, Olivier Garraud, Richard P. Phipps, Neil Blumberg

**Affiliations:** ^1^Department of Pathology and Laboratory Medicine, School of Medicine and Dentistry, University of Rochester Medical Center, Rochester, NY, USA; ^2^Etablissement Francais du Sang Auvergne-Loire, Universite de Lyon, Saint-Etienne, France; ^3^Department of Microbiology and Immunology, School of Medicine and Dentistry, University of Rochester Medical Center, Rochester, NY, USA; ^4^Department of Environmental Medicine, School of Medicine and Dentistry, University of Rochester Medical Center, Rochester, NY, USA; ^5^Department of Medicine, School of Medicine and Dentistry, University of Rochester Medical Center, Rochester, NY, USA

**Keywords:** platelets, transfusion, immune response, transfusion reaction, storage, thrombosis, bleeding

## Abstract

Platelet transfusion has been a vital therapeutic approach in patients with hematologic malignancies for close to half a century. Randomized trials show that prophylactic platelet transfusions mitigate bleeding in patients with acute myeloid leukemia. However, even with prophylactic transfusions, as many as 75% of patients, experience hemorrhage. While platelet transfusion efficacy is modest, questions and concerns have arisen about the risks of platelet transfusion therapy. The acknowledged serious risks of platelet transfusion include viral transmission, bacterial sepsis, and acute lung injury. Less serious adverse effects include allergic and non-hemolytic febrile reactions. Rare hemolytic reactions have occurred due to a common policy of transfusing without regard to ABO type. In the last decade or so, new concerns have arisen; platelet-derived lipids are implicated in transfusion-related acute lung injury after transfusion. With the recognition that platelets are immune cells came the discoveries that supernatant IL-6, IL-27 sCD40L, and OX40L are closely linked to febrile reactions and sCD40L with acute lung injury. Platelet transfusions are pro-inflammatory, and may be pro-thrombotic. Anti-A and anti-B can bind to incompatible recipient or donor platelets and soluble antigens, impair hemostasis and thus increase bleeding. Finally, stored platelet supernatants contain biological mediators such as VEGF and TGF-β1 that may compromise the host versus tumor response. This is particularly of concern in patients receiving many platelet transfusions, as for acute leukemia. New evidence suggests that removing stored supernatant will improve clinical outcomes. This new view of platelets as pro-inflammatory and immunomodulatory agents suggests that innovative approaches to improving platelet storage and pre-transfusion manipulations to reduce toxicity could substantially improve the efficacy and safety of this long-employed therapy.

## Introduction

Since their discovery, platelets have displayed a remarkable development from a pro-thrombotic and pro-hemostatic cell fragment to a surprisingly versatile immune-thrombotic cell. The plethora of findings continues to surprise and excite at the same time. In concordance, an old therapy, i.e., platelet transfusion, either for prophylaxis or acute bleeding has been in transition as well. Initially thought as an “easy fix” for thrombocytopenia, platelet transfusions are now considered a double-edged sword at best. Despite all the controversies, platelet transfusions are a clinical necessity and do save lives every day worldwide. However, the majority of platelet transfusions are administered for prophylactic purposes, and for chronic conditions, rather than acute hemorrhage. It is presently unclear if platelet transfusions are effective in settings of acute hemorrhage. Moreover, the following questions are open and continue to evolve: Do platelet transfusions work the way we think? What are the downsides to this therapy? Some reports already highlight a pro-thrombotic side effect of platelet transfusion, leaving the thrombocytopenic patient, who is already at risk for bleeding with an increased risk of thrombosis and bleeding at the same time.

## Platelets – Novel Findings in Immunity and Inflammation

We briefly summarize a selection of the most relevant findings in regards to an inflammatory and defensive role of platelets from the recent literature.

Platelets are anucleate cell fragments, derived from megakaryocytes and circulate with an average number of 150,000–300,000/μl in humans. Their role in thrombosis and hemostasis has been well described. Their role in inflammation, however, was discovered only relatively recently and has constantly been evolving ever since. From an evolutionary perspective, platelets likely originated from a versatile cell type with both strong hemostatic and defensive cell properties (1, 2). Their role in hemostasis is therefore closely intertwined with their sentinel and inflammatory properties. Activation of platelet surface receptors leads to inside-out signaling, which is followed by integrin activation, which in turn, is crucial for thrombus formation (3). In addition to G-protein coupled receptors (mainly protease-activated receptors, or PARs), platelets also possess immunoreceptor tyrosine-based activation motif (ITAM), e.g., Fc-receptors, glycoprotein VI, and C-type lectin-like receptor 2 (CLEC2). Fc-receptors allow for immunoglobulin and immune complex binding, while GPVI (the major collagen receptor) and CLEC2 are known to be important for vascular integrity in inflammation (4). Toll-like receptors (TLR) are present on platelets as well, although there are conflicting reports about their functionality (5–8). A recent report indicated that platelets were able to discriminate between different LPS isoforms by differential cytokine secretion by mononuclear cells, including IL-6, TNF-α, and IL-8 (9). In addition, platelets express the coxsackievirus and adenovirus receptor (CAR). Of note, coxsackievirus 1 and 3 induced P-selectin exposure and phosphatidyl serine exposure in platelets, albeit independently of CAR. The presence of platelets led to lower coxsackievirus 1 and 3 titers, to less myocardial virus load and to better survival in mice infected with the virus. To a similar conclusion came authors of another recent paper, showing how platelets protect the host during encephalomyocarditis virus (EMCV) infection via TLR7. Platelet depletion led to reduced survival, and transfusion of WT platelets into TLR7-deficient mice improved survival and was accompanied by a drop in platelet count (10). Another virus, which has recently been described to interact with platelets, is the H1N1 influenza virus. During infection with H1N1, platelets show activation of their surface receptors, lipid mediator generation, and release of microparticles. Interestingly, immunized subjects showed circulating immune complexes of virus and IgG, which were able to activate platelets via FcγRIIa. Alternatively, H1N1 virus was able to activate platelets via thrombin generation (11).

Platelets express high-mobility group protein 1 (HMGB1) (12). Upon activation, HMGB1 is translocated from the cytoplasm to the outer plasma membrane. In its extracellular location, HMGB1 has been implicated in inflammatory, proliferative, and migratory processes (13). TLR2, 4, 9, the receptor for advanced glycolation end-products (RAGE) and Mac-1 have been described in the inflammatory actions of HMGB1 (14–16), while ultimately these signals lead to NF-κb activation. While HMGB1 has not been investigated in the context of platelet transfusion, it is entirely conceivable that HMGB1 accumulates during platelet storage and triggers inflammatory sequela upon transfusion.

We and others have demonstrated that although platelets are anucleate cell fragments they express an armamentarium of transcription factors including the NF-κb transcriptional regulatory system, Bcl-3, and PPARγ (17–22). The current concept is that these factors act in non-transcriptional ways in platelets, e.g., by modulating the response to activation, even though they act as traditional transcription factors in megakaryocytes (17, 21).

Although platelets do not possess a nucleus, there is evidence that platelets can synthesize IL-1β in substantial amounts upon activation. In a resting state, platelets contain the pre-mRNA of IL-1β. However, when activated, they synthesize pro-IL-1β protein, which is further processed and ends up as the mature form of the cytokine IL-1β (23, 24). One of the earlier discoveries in platelet biology was the tendency of platelets to “stick” to leukocytes when activated and in generalized inflammatory conditions. All subclasses of leukocytes have been described to adhere to platelets (25–27). However, the majority of studies describe myeloid leukocytes as the platelet-binding partner. The interaction between platelet and leukocyte mainly depends on the P-selectin – PSGL-1, and fibrinogen – αIIbβ3, or directly to GPIb and αMβ2 (28).

The initial contact between leukocyte and platelet is established via platelet P-selectin and leukocyte PSGL-1. On the molecular level, P-selectin binds in a stereospecific manner to the N-terminal region of PSGL-1, by recognizing a motif with tyrosine sulfate residues, fucose, galactose, and sialic residues on a core-2 *O*-glycan (29). Overall, the interaction between P-selectin and PSGL-1 has a very rapid association/dissociation rate, facilitating the rapid capture, tethering, and rolling under high flow and shear conditions (30). Of note, this initial step of tethering and rolling does not require any leukocyte activation signals, but subsequent steps of firm adhesion and transmigration require signaling events that ultimately lead to integrin activation. Specifically, Nef-associated factor 1 (Naf-1, downstream of PSGL-1) is phosphorylated by Src-family kinase (SFK), and leads to Mac-1 (αMβ2) activation. In addition, neutrophils show LFA-1 (αLβ2) activation, while monocytes and lymphocytes show β1 and β2 activation (31, 32). Integrin outside-in signaling via SFK then leads to phosphorylation of proline-rich tyrosine kinase 2 (Pyk2), which is followed by a sustained leukocyte activation with stabilization of the integrin bond and a delayed inflammatory response including NF-κb activation. Furthermore, if circulating as heterotypic aggregates, they can facilitate leukocyte deposition at sites of inflammation and vascular injury and have been described in a plethora of diseases (33–36).

Notably, platelets contain substantial amounts of CD40L (formally known as CD154), a protein with a significant role in T-cell-dependent isotype switching and generation of antibody subclasses by B-cells. Platelets can stimulate neutrophils, T-cells, and endothelial cells via CD40L (37–39). In addition to interacting with leukocytes, platelets closely interact with endothelial cells. Inflammatory conditions are usually accompanied by a certain degree of vascular leakiness. Interestingly, thrombocytopenic patients usually do not bleed with platelet counts above 10,000/μl unless they develop inflammation. This contributory role of platelets to vascular integrity seems to mainly depend on platelet ITAM receptors (4, 40–42). In a similar way, platelets seem to protect the vasculature in tumors and prevent bleeding inside of tumors (43, 44). Surprisingly, platelets have also been found to enhance vascular permeability during inflammation, perhaps due to their secretion of VEGF, serotonin, and other similar factors. These seemingly contradictory results might reflect model, species, and site-specific differences, but it might also depend on a differential release of mediators, specifically serotonin has been shown to be crucial for the induction of vessel permeability by platelets (45). How the differential contribution to vessel integrity and leakiness is regulated *in vivo* remains to be investigated. Examining vascular integrity during dengue virus infection, Hottz et al. found that platelets contribute to vascular leakage by releasing IL-1β-rich microparticles after assembly of nucleotide-binding domain leucin-rich repeat containing protein (NLRP3), and are thus likely to contribute to hemorrhage frequently observed during infection with this virus (46). Other virus-induced hemorrhagic fevers also critically involve platelets (47). Recent studies also highlight the ability of platelets and platelet-like particles to transfer microRNA to leukocytes and endothelial cells. Even though this has not been shown to be the case in an inflammatory setting, it is an interesting mechanism to transfer “long distance” information and contribute to vascular homeostasis (48). An intriguing recent study by Massberg et al. suggested that platelets serve as “first sensors” for endothelial damage and potential invaders. Platelets adhered to and activated neutrophils, which in turn released nucleosomes and serine proteases in order to trigger the intrinsic and extrinsic coagulation pathway. This localized promotion of thrombosis by platelets and neutrophils in order to trap invaders in the microcirculation is a novel example of how closely intertwined innate defense mechanisms and thrombotic processes are (49, 50).

One of the most striking recent findings is how neutrophils produce extracellular traps, or “neutrophil extracellular traps (NETs)” in order to cause thrombosis and thereby trap invading microbes. While neutrophils are the major inducer of NETs by ejecting their nucleus, there is emerging evidence that platelets assist neutrophils in this process: platelet β-defensin-1 released after stimulation with *Staphylococcus* α-toxin was found to be critically involved in the formation of NETs under flow conditions *in vitro* and *in vivo* (51). Earlier, an elegant study by Clark et al. showed that TLR4 on platelets binds to circulating LPS and mediates neutrophil–platelet binding with subsequent neutrophil activation and NET formation in mice and humans. The authors were able to show that NET formation helped trapping bacteria. Similar to the study mentioned above by Massberg et al., platelets function as a “sensor” for circulating LPS and facilitate neutrophil activation (52). Of note, platelets were also critically involved in NET formation in a mouse model of transfusion-related acute lung injury (53, 54). A somewhat under-recognized cell organelle in platelets has recently been shown to contribute to the inflammatory response by platelets: by releasing mitochondria, either free or coated by microvesicles, they provided the substrate for secreted phospholipase A_2_-IIa (PLA_2_-IIa), which in turn produces lysophospholipids, fatty acids, and mtDNA to activate leukocytes (55).

## Platelet Storage and Inflammation

**Figure 1:**
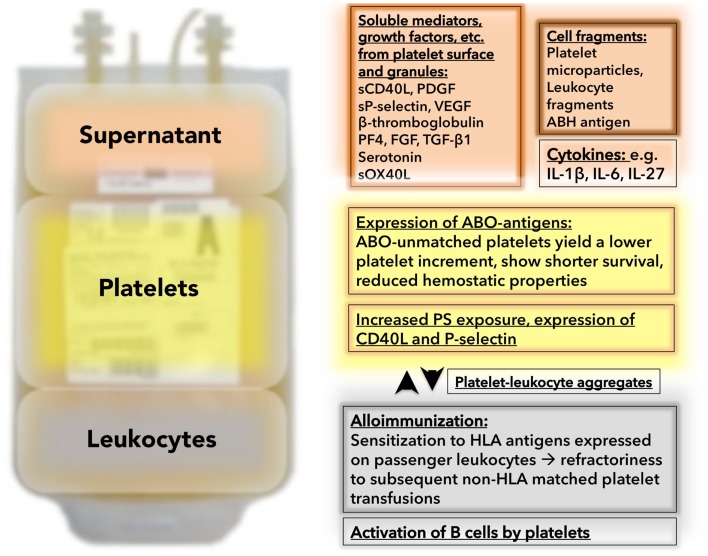
**Unwanted passengers and events during platelet storage: Besides platelets, platelet concentrates contain “unwanted passengers” in the form of soluble mediators in the supernatant (in the case of unwashed platelet concentrates – which are still the routine product in virtually all US hospitals) and leukocytes (in the case of non-leukoreduced platelet concentrates – which are still an available product in some US hospitals)**. The supernatant (red background) contains substances which are secreted or shed into the plasma during storage containing soluble mediators, like sCD40L, PDGF, or sP-selectin. In addition, cytokines and cell fragments can be found. The platelet themselves (yellow background) change during storage. They become hyporesponsive (not depicted), and degranulate with increased expression of alpha granule contents (e.g., P-selectin). Platelets express ABO antigens, which lead to suboptimal results and complications if transfused into ABO-unmatched recipients. A marker for platelet activation is platelet leukocyte aggregate formation (white background). With prolonged storage, the number of platelet leukocyte aggregates increase. Leukocytes in the stored bag (gray background) release cytokines, which can induce febrile transfusion reactions. Passenger leukocytes lead to sensitization to HLA antigens with subsequent refractoriness.

Prolonged storage leads to changes in platelets mostly associated with platelet activation. Contents of the alpha granules are secreted which leads to a gradual increase in P-selectin and CD40L membrane expression. Consistently, the number of platelets attached to leukocytes increases over time during storage (56). A substantial amount of P-selectin and CD40L are shed into the plasma as soluble P-selectin (sPS) and sCD40L, respectively (57, 58) (Figure 1). CD40L and its interaction with neutrophils and endothelial cells have been implicated in the development of non-antibody-mediated transfusion-associated acute lung injury (TRALI) (39). Of note, platelets act in concert with T-cells to mediate B-cell stimulation and function as a “first wave” of B-cell activation (37). Specifically, after a 3-day incubation with platelets, B cells showed an increased production of IgG1, IgG2, and IgG3, but not IgG4, IgA, or IgM (37). Interestingly, activation of dendritic cells by platelets has proven to be independent of sCD40L. While platelets do stick to dendritic cells, dendritic cell activation is mediated by nucleotides like ADP and ATP (59). Platelet CD40L has also been involved in host defense against *Listeria monocytogenes* in a mouse model (60). In addition to its role in inflammation, platelet CD40L has also been implicated in more traditional roles of platelets such as thrombus formation and thrombus stabilization (61). In addition to P-selectin and CD40L, multiple other platelet alpha and dense granule substances are found in the supernatant: e.g., β-thromboglobulin, platelet factor 4, and serotonin (62).

Stored platelets also exhibit an increase in phosphatidylserine (PS) exposure (Figure 1), which can be assessed via annexin V binding. Of note, the degree of platelet activation does not correlate with P-selectin expression, platelet count increment, or function *in vivo*. P-selectin-expressing platelets that were transfused into primates and rabbits rapidly lost their surface P-selectin but continued to function and to circulate *in vivo* (63–65).

Platelet microparticles (PMP) are released from platelets upon activation or apoptosis. Platelet concentrates contain PMPs in the supernatant, reflecting activation during collection, and/or storage (Figure 1). Apheresis collection is associated with less PMP formation when compared to platelets separated from whole blood donations (66, 67). PMPs have been shown to be critically involved in inflammatory conditions like rheumatoid arthritis (68). Infusion of activated platelets and microparticles causes early atherosclerotic lesions (fatty streaks) and exacerbate atherosclerosis in mice and diabetic patients (69–71). Furthermore, PMPs enable platelets to transport RNA, cytokines, or chemokines to other cells and tissues, as they are small enough to exit the circulation and to enter the surrounding tissues. This has been shown for RANTES (72), IL-1α (68), IL-1β (24, 73), PPARγ (74), retinoid X transcription factor (74), and CD40L (75, 76), albeit not under transfusion conditions. Human cytomegalovirus (HCMV) activates platelets via TLR2 and triggers ADP release and subsequent platelet leukocyte aggregate formation *in vitro* and *in vivo*, which might explain how this virus might contribute to atherosclerotic lesion formation (77).

We were recently able to show that the composition of microparticles can be altered by genetic engineering. Using lentiviral technology, we introduced green fluorescent protein (GFP) and increased PPARγ expression in megakaryocytic cell lines and primary megakaryocytes. Platelets and microparticles generated by modified megakaryocytes were internalized by a monocytic cell line and altered the expression of a target protein of PPARγ. This is a potentially useful tool in altering and investigating transcellular communications (78). To what extend PMP contribute to the hemostatic properties of platelet transfusions has not been yet investigated. Moreover, PMPs function as messengers to convey pro-inflammatory information to endothelial cells and leukocytes. No study has investigated the pro-inflammatory role of PMPs after transfusion yet, but it seems likely that there are some systemic effects to the recipient. Stored platelets also contain minute fragments of leukocytes that have been involved in HLA-sensitization and platelet refractoriness in pre-clinical studies (79) (Figure 1).

Adding some clinical relevance to the aforementioned pre-clinical *in vitro* and lab findings, we were able to show that washing red cells and platelets (i.e., removing the supernatant) is associated with less inflammatory response in high-risk pediatric populations compared to non-washed platelet transfusions (80). Furthermore, the patients in the study-arm needed fewer transfusions and there was a trend to lower mortality in the study-arm (washed units). Larger clinical trials are needed to determine if this finding is clinically significant (80).

## Transfusion Reactions and Immunomodulation

Although platelet transfusions are generally well-tolerated, they cause more transfusion reactions than any other blood product (81). The adverse events range from simple allergic reactions to severe anaphylactic reactions, febrile-non-hemolytic reactions, transfusion-associated sepsis, and TRALI. Several strategies have been shown to reduce the rate of transfusion reactions, e.g., leukocyte reduction, washing, and ABO matching. As described above, stored platelets release sCD40L and accumulate CD40L on their surface. In fact, platelet concentrates from apheresis and whole blood collection demonstrated the highest sCD40L concentrations compared to all other blood products (39). Platelet concentrates involved in TRALI had significantly higher sCD40L levels compared to uninvolved ones. *In vitro* sCD40L was able to prime the PMN oxidase rapidly suggesting that sCD40L could be critically involved in non-antibody-mediated TRALI (39). But, its role might not be limited to that; earlier data from our group suggested that sCD40L could induce febrile transfusion reactions by activating cyclooxygenase-2 and thereby producing prostaglandin E_2_ (82). Of note, the concentrations that were required to produce PGE_2_ *in vitro* are easily met and even exceeded by one order of magnitude after transfusion of unwashed platelets. In addition, cytokine accumulation (e.g., IL-1β, IL-6, IL-27) and accumulation of soluble OX40 ligand in the stored platelet bag has been shown to contribute to febrile non-hemolytic reactions (83, 84) (Figure 1). Washing and leukoreduction was protective and helped to reduce the incidence of febrile transfusion reactions (85–87). In addition to sCD40L, cytokines, and sPS, platelet-derived growth factor (PDGF) accumulates during platelet storage (88). Moreover, other growth factors like VEGF, FGF-2, and TGF-β1 have been shown to accumulate during platelet storage (89–91) (Figure 1). Upon transfusion into patients with (hematologic-) malignancies, these growth factors may promote cancer growth and antagonize growth factor-targeted therapies (90). Overall, there is good reason to believe that the number of described soluble mediators is not complete; as a recent study showed the presence of 1048 proteins in the supernatant, including 69 membrane proteins (10 had been shown to be shed from platelets before). However, this data warrants further validation under storage conditions since platelets were activated with agonists instead of activating them by prolonged storage (92). We previously investigated the platelet proteome under storage conditions, using mass spectrometry and found that 117 proteins changed during storage conditions. Notably, 22 out of 117 proteins were previously described in platelets and two-thirds of these 22 were associated with alpha granules, supporting degranulation as one of the major events during storage (93).

In an intriguing study, Yazer et al. investigated if a febrile non-hemolytic transfusion reaction renders the patient more susceptible for the development of alloantibodies to subsequent red blood cell products, and found a significantly higher rate of sensitization in the study-arm (94). This result corroborated recent findings in animal models (95, 96) and is presumably due to cytokines in the platelet unit that are high enough to induce a febrile transfusion reaction and generate a humoral immune response which predisposes the recipient to more effective antibody production (97). The idea that transfusions could alter the immune system of the recipient other than through alloimmunization stems from the discovery that allogeneic transfusions enhanced kidney graft acceptance in transplant recipients (98). Shortly afterwards, an association between colorectal cancer recurrence and transfusion was noted and confirmed in retrospective studies (99–101). In concordance was another subsequent finding that transfusions are associated with infections perioperatively in cancer patients (102). Although all of the above mentioned initial work on transfusion-mediated immunomodulation was done on red blood cell transfusions, it is likely that soluble mediators that accumulate in platelet concentrates have an immunomodulatory effect upon transfusion into the recipient as well. Specifically, sCD40L and similar mediators could alter host defense in a way that tumor defense is impaired by shifting the immunity toward a type-2 immune function (103, 104). Our group demonstrated that sCD40L stimulates PGE_2_ production and induces COX-2 (58, 82, 105, 106). Another possible mechanism may be that sCD40L functions as a proliferation and survival factor for the circulating leukemic cells. It is likely that washing platelet concentrates before transfusions improves survival in leukemic patients (107, 108). We were recently able to replicate our previous findings in younger patients with acute myeloid leukemia with a striking survival benefit of almost 100% in patients with favorable risk AML on a washed transfusion protocol and a short-term mortality of nearly 0% in patients with any type of AML in the treatment group (washed protocol, unpublished observations). Washing the platelet concentrate is an inexpensive and relatively easy way to get rid of the supernatant. It goes along with a mild activation and a minor loss of platelet numbers prior to transfusion. However, these modest downsides are disproportionate as compared to the potential benefits to patients in terms of survival.

Interestingly, the rate of TRALI is highest in platelet transfusions. Although it has never been convincingly demonstrated that transfused platelets are causally related to the development of TRALI, a recent discovery linked recipient platelets to the pathogenesis in antibody-mediated TRALI. Aspirin treatment and platelet depletion prevented TRALI in a mouse model (54). Furthermore, two papers linked NETs to the pathogenesis of TRALI and one of them linked the occurrence of NETs directly to platelets (53, 109).

## ABO Matching for Platelet Transfusions

The ABO blood group system is still the most important system in transfusion medicine since it was first discovered by Karl Landsteiner roughly a century ago. ABO antigens are both integral to and passively adsorbed onto the red blood cell surface. The discovery that platelets also express ABO antigens was made in the 1950s (110). More recently, they have been localized on integrins αIIbβ3 and α2β1 (111, 112). One of the earliest clinical discoveries was that non-ABO-matched platelets transfusions yield a lower platelet count increment (113). This was further substantiated in multiple randomized and observational studies; in addition ABO-matched platelet transfusion protected better from bleeding and led to less frequent refractoriness (114–116). *In vitro*, co-incubation of A, B, or AB platelets with O plasma with differing titers of anti-A and anti-B inhibited platelet aggregation and was also associated with other anticoagulant properties, delivering a potential explanation for the reduced hemostatic activity of non-ABO-matched platelet transfusions (117). The lower platelet increment is speculatively due to clearance of antibody-coated platelets and platelets coated with immune complexes by phagocytic cells. We showed previously, that circulating ABO immune complexes that were isolated from patients after non-ABO-matched transfusion were able to adhere to platelets via the Fc-receptor and complement receptors, cC1q and gC1q. In addition, *in vitro* formed ABO immune complexes showed the same ability to bind platelets via the aforementioned receptors. Inhibition of the receptors with blocking antibodies reduced the amount of bound immune complexes by 67–99%. Together these findings provide an additional potential mechanism for platelet clearance after non-ABO-matched transfusion and overall for the importance of ABO-matched transfusions clinically (118).

In a cohort trial investigating the effect of ABO matching platelets in patients undergoing cardiac surgery, we showed that patients who received at least one ABO-mismatched pool of platelet had significantly longer hospital stays, more days of fever, and more RBC transfusions. Notably, recipients of ABO-identical platelets had one-quarter the mortality, fewer mean days of antibiotics and hours in the ICU than patients receiving ABO-mismatched transfusions. Maybe due to the smaller number of patients, these latter differences were not significant (119). Our institution switched to universal ABO-matched platelet and cryoprecipitate transfusion in 2005, and this approach led to improved clinical outcomes and reduced transfusion requirements in surgical patients (120). Furthermore, this approach has also proven feasible from a blood management and administrative standpoint (121). Table 1 summarizes all findings from trials that were performed by our group, at our institution with regards to ABO matching and platelets transfusions.

**Table 1:** **Studies investigating the role of ABO-group matching in platelet transfusions**.

Author	Study type/question	Result	Year	Journal	Reference
Heal et al.	*In vitro* study investigating the role of circulating immune complexes after ABO-non-identical transfusion	Circulating immune complexes adhere to platelets via Fc-receptor and complement receptors and provide a potential mechanism for platelet clearance	1996	Vox sanguinis	(118)
Blumberg et al.	Retrospective cohort-study investigating patients undergoing cardiac surgery	Patients who received ABO-mismatched platelets had a longer hospital stay, more fever, and more RBC transfusion. Furthermore, there was a trend toward a 75% reduction in mortality with ABO-identical platelets	2001	Transfusion	(119)
Refaai et al.	Retrospective analysis of non-ABO-identical platelet transfusion and the effect on transfusion requirements and other clinical parameters	ABO-identical transfusions might lead to lower transfusion requirements and better clinical outcome	2011	Vox sanguinis	(120)
Henrichs et al.	Feasibility trial, designed to answer if uniform ABO-identical platelet transfusion is doable in a tertiary care hospital setting	97% of patients received ABO-identical platelets. There was an unexpected reduction in febrile and allergic reactions. In addition, there was a reduction in RBC alloimmunization and HLA platelet requirements	2012	Transfusion	(121)
Refaai et al.	*In vitro* study investigating the effect of anti-A and anti-B on platelet function and clot formation	Anti-A and anti-B inhibit platelet aggregation and reduce clot formation in various *in vitro* assays	2013	Transfusion	(117)

*The table summarizes the *in vitro* and clinical studies by our group at the University of Rochester investigating the role of the ABO blood group system in platelet transfusions*.

## Alloimmunization to HLA

Patients who receive frequent platelet transfusions sometimes become less responsive to platelet transfusion, a phenomenon termed “refractoriness.” There are immune-mediated mechanisms of platelet refractoriness and non-immune-mediated mechanisms. Overall, the non-immune platelet removal is more common than immune-mediated removal (122). However, in light of the focus of this review article, we will focus on the immune-mediated mechanisms. The most common reason to develop immune-mediated platelet refractoriness is the development of antibodies to foreign HLA A, B (class I MHC), which are expressed on platelets and most other cell types, or the development of platelet-specific antibodies. Interestingly, in case of the sensitization to HLA antigens, transfusion of leukocyte containing blood products is associated with a higher rate of immunization, and consistently, universal leukoreduction leads to a significant reduction in platelet refractoriness (123) (Figure 1). This along with others lead to the conclusion that transfusing a small amount of leukocytes with red blood cells or platelets is more immunogenic than the transfused platelets themselves. Our institution’s policy is to transfuse only leukoreduced and ABO-identical platelets, and our internal data suggest that this leads to fewer incidences of platelet refractoriness (<1% of patients). One potential solution when alloimmunization has occurred and refractoriness is evident is to expend the money necessary and engage in the time-consuming process of finding an HLA-matched donor. This can be facilitated by HLA-matching or by a test that determines the antibody specificity and selects donors based on their lack of the corresponding antigens (124).

## Platelet Transfusion and Thrombosis

Inflammation and thrombosis are closely intertwined; it is therefore conceivable that platelet transfusions not only lead to inflammation, but also thrombosis. Indeed, there is evidence, mainly coming from an observational study, that transfusions are associated with thrombosis. A retrospective study investigated the associations between transfusions and venous thromboembolism, arterial thromboembolism, and mortality in hospitalized patients with cancer. All three outcomes were significantly more common in patients that received platelet transfusion (125). However, more studies, preferably randomized, controlled trials are needed to show this association is indeed causal. More data is available for red blood cell transfusions (126, 127), while overall clinical data on this subject is still rather scarce.

It is one of the issues of platelet transfusions that the hemostatic potential of the stored platelets decrease over time in the storage bag. On the other hand, the amount of soluble pro-inflammatory and pro-thrombotic mediators in the supernatant increases. These mediators presumably partially compensate for the loss of platelet function, but it is plausible that they contribute to thrombosis as well. Platelet-derived microparticles and sCD40L have been shown to be involved in thrombosis under pre-clinical, experimental conditions and are most likely amongst the culprits that mediate thrombosis (128–131).

Overall it appears, as if fresher platelets (<3 days of storage) are preferable due to reduced cytokine load and are likely less pathogenic. More clinical studies are needed to evaluate the hemostatic improvement and/or impairment, as well as the thrombotic risk as a function of storage time.

## Author Contributions

MS and NB wrote a draft of the manuscript and revised it critically for important intellectual content. MR, SS, JH, and RP provided important feedback and revised the manuscript critically for important intellectual content. All authors approved the final version of the manuscript.

## Conflict of Interest Statement

The authors declare that the research was conducted in the absence of any commercial or financial relationships that could be construed as a potential conflict of interest.

## Abbreviations

NET, neutrophil extracellular trap; PMP, platelet microparticles; sPs, soluble P-selectin.
